# Three-Dimensional Printed Stimulating Hybrid Smart Bandage

**DOI:** 10.3390/s25165090

**Published:** 2025-08-16

**Authors:** Małgorzata A. Janik, Michał Pielka, Petro Kovalchuk, Michał Mierzwa, Paweł Janik

**Affiliations:** Institute of Biomedical Engineering, Faculty of Science and Technology, University of Silesia in Katowice, 41-205 Sosnowiec, Poland; michal.pielka@us.edu.pl (M.P.); petro.kovalchuk@us.edu.pl (P.K.); michal.mierzwa@us.edu.pl (M.M.); pawel.janik@us.edu.pl (P.J.)

**Keywords:** smart, mobile, IoT, wearable, embedded system, wound healing

## Abstract

The treatment of chronic wounds and pressure sores is an important challenge in the context of public health and the effectiveness of patient treatment. Therefore, new methods are being developed to reduce or, in extreme cases, to initiate and conduct the wound healing process. This article presents an innovative smart bandage, programmable using a smartphone, which generates small amplitude impulse vibrations. The communication between the smart bandage and the smartphone is realized using BLE. The possibility of programming the smart bandage allows for personalized therapy. Owing to the built-in MEMS sensor, the smart bandage makes it possible to monitor work during rehabilitation and implement an auto-calibration procedure. The flexible, openwork mechanical structure of the dressing was made in 3D printing technology, thanks to which the solution is easy to implement and can be used together with traditional dressings to create hybrid ones. Miniature electronic circuits and actuators controlled by the PWM signal were designed as replaceable elements; thus, the openwork structure can be treated as single-use. The smart bandage containing six actuators presented in this article generates oscillations in the range from about 40 Hz to 190 Hz. The system generates low-amplitude vibrations, below 1 g. The actuators were operated at a voltage of 1.65 V to reduce energy consumption. For comparison, the actuators were also operated at the nominal voltage of 3.17 V, as specified by the manufacturer.

## 1. Introduction

Increasing treatment effectiveness is a socially relevant issue. Acceleration of the treatment process makes it possible to reduce its cost and the time of social exclusion. Society’s expectations concerning the availability of health technologies [1], including those that can be used at home, are constantly increasing. Therefore, it is important to produce new technologies as well as to reduce the costs of their potential production, or produce alternative, cheaper counterparts of the popular and hard-to-access solutions [2]. The development of wearable sensor technologies that can be produced using sewing machines [3] and smart textile biosensors [4] is in line with this concept. One of the social problems is the treatment of wounds, including chronic ones, and pressure sores. The risk group in which chronic wounds may occur includes people with metabolic diseases such as diabetes [5]. It is, therefore, estimated that in the world population, about a billion people are in this risk group [6]. In this regard, in recent years, there has been a dynamic development of the concept of the so-called smart bandages. These are complex structures that make it possible to monitor some selected body functions or distribute drugs [7,8,9]. One of the applications of this type of solution is wound monitoring using flexible bioelectronic bandages [10,11], flexible breathable materials [12], or inkjet-printed smart bandages [13]. In turn, in the process of wound treatment, it is beneficial to use cheap single-use solutions [13,14,15]. Wound healing can also be accelerated by using electric stimulation [11], bioelastomers [16], and gel dressings [17]—which make it possible to adjust the pressure in the wound [18]—or electroactive dressings [19]. The dynamic development of the smart bandage concept is supported using 3D printing technology. In this respect, research is conducted on the application of, among others, hydrogels [20,21]. Three-dimensional printing also allows for the production of complex and electronically controlled structures that use microneedles to provide drugs [22]. Extrusion-based printing methods are also used to produce topical skin applications [23]. Currently, smart bandages are complex systems that allow for data transmission and wound monitoring via smartphones [10,24,25,26,27]. In the context of treating wounds, including chronic ones, it is worth mentioning the use of vibrations of various frequencies and amplitudes [28]. Vibrations are used as a mechanotransductive cell engineering tool for both in vitro phenotypic control and in vivo regenerative therapy [29]. Vibration wound therapy can accelerate the foot wound healing process [30]. Moreover, the use of vibration platforms makes it possible to accelerate diabetic neuropathy foot ulcer healing in patients diagnosed with diabetes [31]. As far as wearable solutions for wound treatment are concerned, it may be beneficial to use high-frequency (>20 Hz) vibrations with small amplitudes. This is indicated by research on stimulating macrophage growth. Macrophages are essential for the efficient healing of various tissues. The use of vibrations with a frequency of 100 Hz and the smallest of the tested acceleration values of 0.15 g resulted in the highest increase in the number of macrophages [32]. These data indicate that the solutions for wound treatment using high-frequency and low-amplitude vibrations (LIV) have potential for practical applications. Experiments with diabetic mice indicated the impact of LIV (0.4 g at 45 Hz) on wound healing [33]. LIV increased, among other things, angiogenesis and granulation tissue formation, accelerated wound closure and re-epithelialization, and increased macrophage accumulation [32].

This article presents the concept of a smart bandage system using vibration actuators. The actuators generate low-amplitude vibrations, below 1 g, in the frequency range below 200 Hz. When designing the system, the following requirements were taken into account: low production costs (standard electronic components), the possibility of using single-use and universally accessible components, as well as cheap manufacturing technology (3D printing). In addition, the solution makes it possible to scale the wearable part. These technological features used in one system confirm its high innovation. In addition, the use of many miniature actuators controlled in a programmable manner, allowing for modeling vibrotherapy cycles, introduces new therapeutic possibilities. In turn, the proposed concept of a hybrid dressing combines classic methods of wound treatment—including chronic wounds—with high technologies, in particular wearable electronics and mobile technologies, which are part of the IoT.

In the present article, it was assumed that oscillators would generate vibrations at a similar level so that the distribution of oscillations within the smart bandage would be homogeneous. The solutions described in the literature are usually based on one actuator. In the present article, low-amplitude vibrations are generated by several actuators. Thus, the possibility of controlling many actuators and the vibration distribution provides new biomedical applications.

## 2. Materials and Methods

### 2.1. Measurement Equipment

The solution presented in this article is a concept of a smart bandage intended for vibrotherapy. The prototype created in the laboratory was subjected to a series of tests to verify its technical properties. The use of smart bandages in clinical practice requires further research and the adaptation of used components, e.g., wiring, or the development of an integrated wearable solution (combining electronics and the battery).

The skeletal smart bandage (SSB) was designed as a modular system, which allows for scaling its functionality and integrating modules in various configurations. SSB is an active system that generates vibrations. When the vibrations are placed within the wound area, they can increase blood supply and, in this way, may have a positive effect on accelerating the healing process [28].

**Figure 1:**
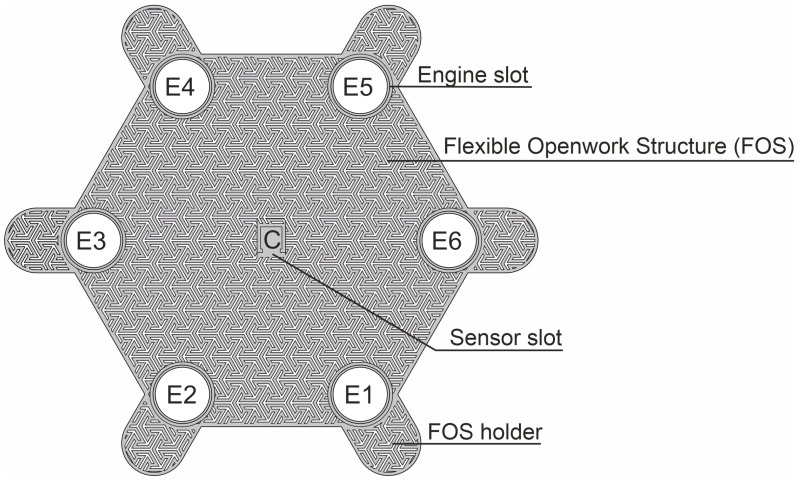
Openwork bandage structure.

The first of the system modules is a flexible openwork structure (FOS), presented in Figure 1, which is the SSB’s mechanical base. The openwork structure is a skeletal frame, within which vibrations generated by actuators are transferred. The FOS does not block the access of air to the surface on which it is placed, owing to which it can be combined with classic dressings, creating a hybrid structure. The FOS is made in 3D printing technology, which allows for the quick modification of its mechanical structure and the adjustment of the shape to individual needs, i.e., personalization. The FOS contains slots in which the actuators (mini-vibration engines) and the sensor (accelerometer) are placed, which is described in more detail later in this article. This openwork structure concept allows it to be used as a replaceable element, which in the case of dressings is particularly important. Figure 1 shows the openwork structure in the shape of a hexagon with a side length of 45 mm and a thickness of 0.5 mm, designed for 6 actuators (E1–E6). The openwork structure also includes a slot for the accelerometer, which is located in the middle of the structure and is marked as C. The FOS also has holders (FOS Holder), which can be used for SSB attachment, for example, to a traditional dressing, using an adhesive tape or standard clips.

**Figure 2:**
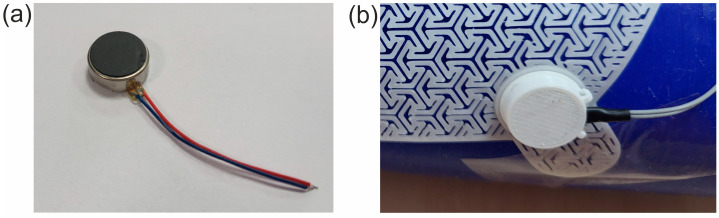
Images of (**a**) vibrating engine (actuator); (**b**) printed housing of the MT24 vibrating engine.

MT24 miniature vibration engines with a diameter of 10 mm and a height of 3.7 mm were used as vibration generators in the SSB. The actuators were placed in E1-E6 slots. Similar actuators are used for quiet notifications in mobile devices. According to the SSB concept, the number of vibration actuators used can be variable and selected depending on the needs, which allows for scaling the smart bandage structure. Each of the actuators was locked in a designed housing and then placed in the appropriate mounting sockets in the openwork structure. The miniature MT24 actuators are presented in Figure 2a. The vibrating engine placed in the housing and the enlarged fragment of the openwork structure made in 3D printing technology are shown in Figure 2b.

The applied actuators are safe to use because both the drive itself and the rotating mass are located inside the metal (factory) housing. From the outside, there is no access to any rotating element. The printed housing, however, performs the function of the actuator fastening element. In the case of damage to the printed housing, there is no danger of exposure of the wound to the vibrating elements. In addition, the housing is airtight. Therefore, sterilization can be performed using standard agents, e.g., based on octenidine. The concept also assumes that due to low costs, the openwork structure (FOS) can be replaced after each use, and so sterilization may not be necessary.

SSB is to be placed on the applied specialized dressing, which is designed to, for example, absorb blood and exudate from the wound. It is crucial that the SSB covers the wound area, whereas the actuators are outside this area. Only the openwork structure, which can be single-use, is to be located directly above the wound.

**Figure 3:**
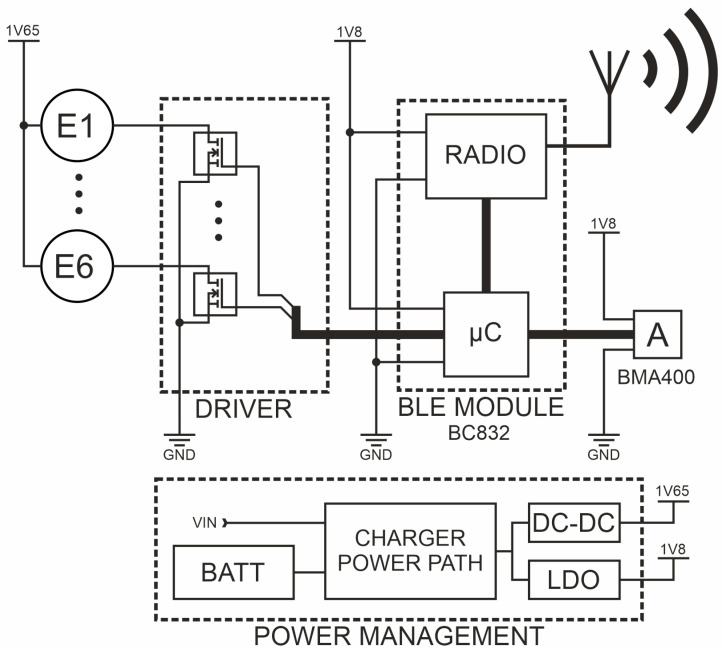
The architecture of central module (CM, controller).

Another SSB function block is the programmable central module (CM), which is a built-in smart bandage system. The CM controls the actuators (MT24) and the measurement system. Wiring and electronics can be disassembled and used in another openwork structure. The central module has reduced architecture [34,35,36,37], i.e., the microcontroller is built into the BC832 radio module [38] with a chip implemented in SOC technology (System on Chip)—NRF52832 [39]. Vibrating engines E1 to E6 are attached to the CM via transistor keys (DRIVER), which are also output buffers of the BC832 module. An ultra-low-power acceleration sensor—BMA400 [40]—is also attached to the central module. It makes it possible to monitor the work cycles of individual engines and to perform the calibration procedure. Communication between the NRF52832 chipset and the BMA400 sensor was implemented using the I2C bus. The accelerometer, used for both performing measurements and carrying out the actuator calibration procedure, was placed in the central slot marked as C. The central module has a built-in energy management block (Power Management). This block contains both the system for controlling charging and power flow (Power Path Charger) from the battery (BATT) or an external source (attached to the VIN), as well as the voltage converter (DC-DC) and voltage stabilizer (LDO). The architecture of electronic circuits is presented in Figure 3.

The measurement data from the accelerometer were transmitted by radio (BLE—Bluetooth low energy), whereas data acquisition was carried out on a smartphone. An application was also implemented on the smartphone that allowed for controlling the SSB operating modes.

**Figure 4:**
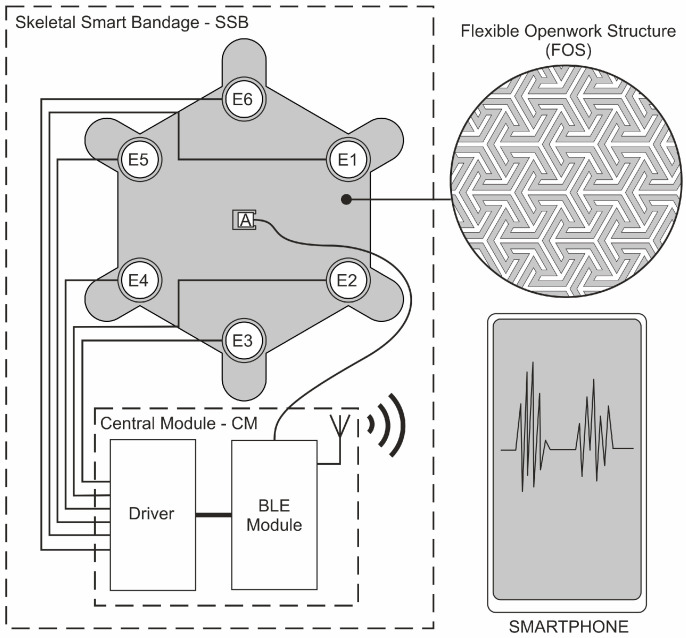
System components: skeletal smart bandage and smartphone.

**Figure 5:**
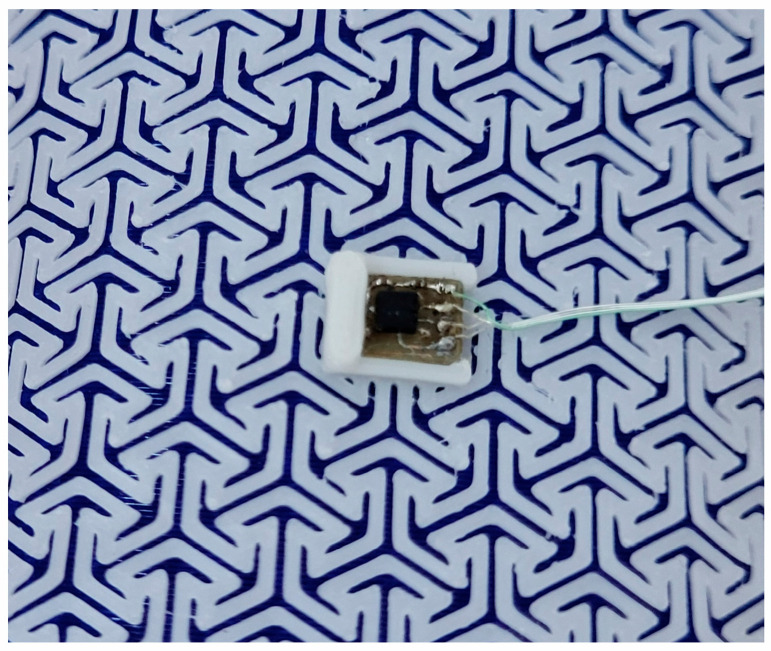
View of the accelerometer placed in the slot in the central part of openwork structure.

The diagram of vibration actuators and electronics implemented in the SSB is presented in Figure 4, and Figure 5 presents a miniature PCB containing BMA400 placed in the central slot of the openwork structure.

### 2.2. Verification of Wearable System

**Figure 6:**
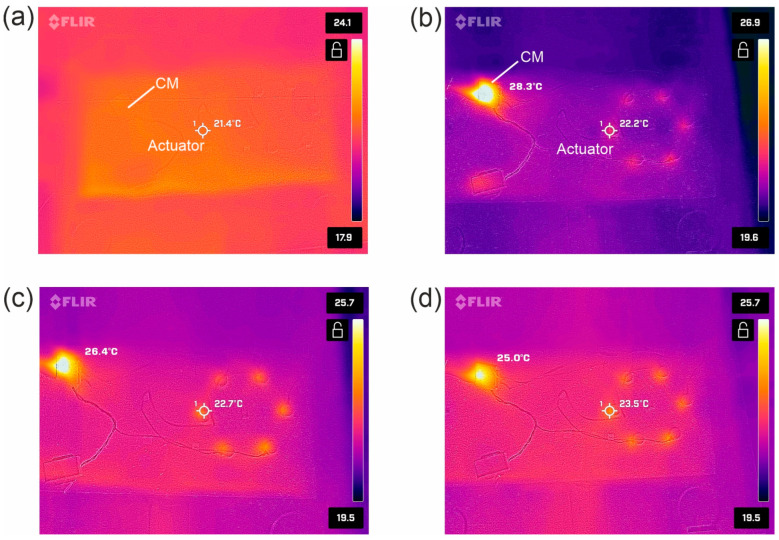
Images of the tested system from the thermal imaging camera: (**a**) before starting work; (**b**) after 15 min of working; (**c**) after 30 min; (**d**) after 1 h.

Wearable solutions should be characterized by work stability and provide thermal comfort for users. In order to verify these properties, a measuring cycle was carried out during long-term operation and with high load of the controller. The test was performed in the darkroom to reduce external IR radiation. Data from the literature show that individual vibrotherapy sessions last on average from 5 to 30 min [28,33]. To demonstrate the stability of the system operation, a 60 min test was carried out, during which individual actuators were activated sequentially every 2 s and their working time was also 2 s. Each actuator was activated at the maximum filling of PWM and a voltage of 3.17 V. In addition, the controller was attached to the charger while working. This increased its load because it also recharged the battery. The test was carried out on a flat surface made of PE Technical Foam, which is a thermal insulator. As a result, the impact of the substrate on temperature measurement was limited. The measurements were made every 2.5 min using a miniature thermal imaging camera (Flir One Gen 3). During each measurement—apart from the first one (determining the temperature of the system before starting work, Figure 6a)—two thermal images were recorded, from which the temperatures of the controller and one selected actuator were read independently. Examples of images are presented in Figure 6.

**Figure 7:**
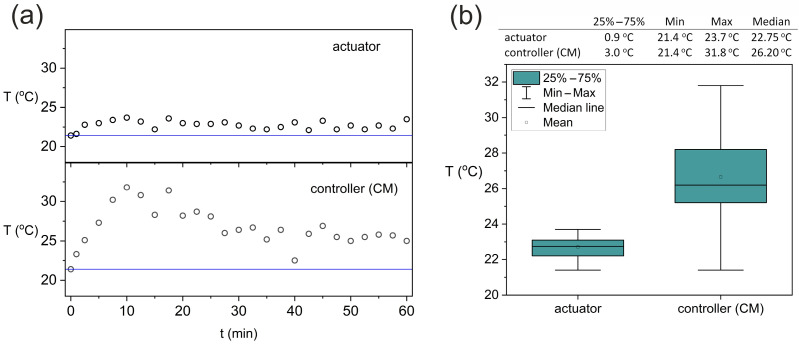
Changes in the temperature of the system (SSB) during operation: (**a**) dependence of the temperatures of the actuator and controller (CM) on working time; (**b**) box-and-whisker plots.

The results of the temperature changes during the system operation are shown in Figure 7a, where the straight line refers to the temperature of the disabled system (21.4 °C). The mean temperature of the actuator during one-hour measurement was 22.7 ± 0.6 °C, and for the controller it was 26.7 ± 2.5 °C. The differentiation of the temperature in both cases was small and amounted to 2.6% for the actuator and 9.6% for the controller. The analysis of the obtained curves shows that the actuators reach the maximum temperature of approx. 24 °C, which is approx. 3 °C above the surface (insulator) temperature. After starting the system, it can be observed that the temperature of the actuator oscillates around 24 °C.

In turn, the temperature of the controller increases to a maximum value of approx. 32 °C. In the initial phase of the system operation, there is a relatively high increase in the temperature of the controller. After about 10 min, the temperature begins to gradually decrease until stabilizing and oscillates around about 25 °C. Such characteristics are related to the fact that in the initial phase, the battery was recharged, and after about 10 min, the charging controller built into the CM gradually reduced the battery charging current. After about 30 min, the cyclically working actuators were the main load for the CM controller. Therefore, it can be assumed that when the system is operating powered only by the battery, the controller reaches a temperature of approx. 4 °C above the substrate temperature.

Figure 7b presents the box-and-whisker plots, which visualize the basic descriptive statistics of the temperature distribution during one-hour operation of the system. The test carried out under laboratory conditions also took into account the non-recommended application, i.e., the operation of the system during charging. The 60 min test indicates the stable operation of the system.

Moreover, taking into consideration the fact that the openwork structure and actuators are not placed directly on the skin and the electronics of the CM controller can be additionally insulated in the housing, it can be assumed that the system will be thermally safe for the user.

**Figure 8:**
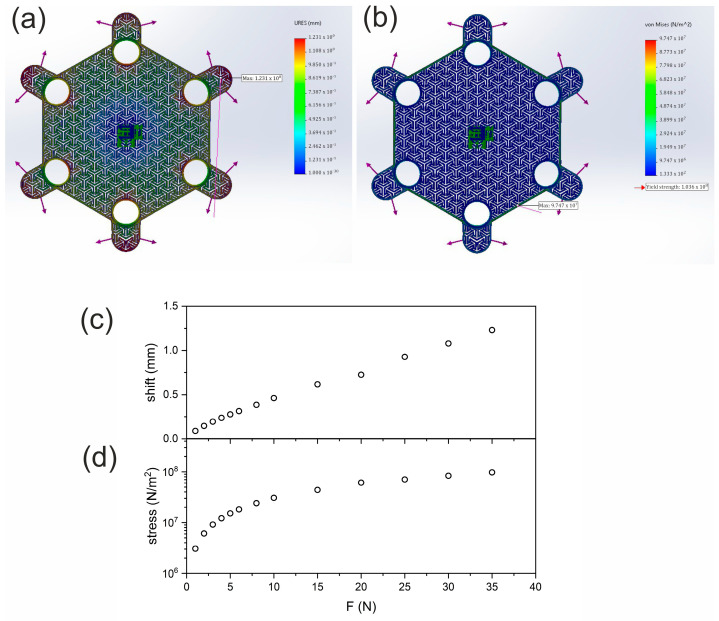
Results of the mechanical strength simulation of FOS made of nylon: (**a**) visualization of the impact of the applied force (35 N) on structure stretching; (**b**) visualization of the impact of the applied force (35 N) on stress; (**c**) a graph of the dependence of the structure shift on the applied stretching forces; (**d**) dependence of the structure stress on the applied stretching forces.

In order to verify the mechanical strength of the openwork structure, simulations were performed in the SolidWorks 2024 SP 1.0 program. It was assumed that the smart bandage is simultaneously stretched in six directions, according to the location of the FOS holder. However, the point at which the stretching occurs is in the center, where the accelerometer is located. Figure 8 shows a visualization of applying the stretching forces to the FOS and their impact on the mechanical parameters of the structure.

For the simulation, the stiffest structure was chosen, i.e., made of nylon, whose parameters are directly implemented in the selected simulation environment. Figure 8 presents the maximum shift/stretch (Figure 8c) and stress (Figure 8d) generated in the structure after force application. The structure was broken when each of the six FOS holders was loaded with 40 N force. Therefore, the whole structure may break up after applying a total of 240 N. FOS is broken when the stretching exceeds 1.23 mm and stress 10^8^ N/m^2^.

According to Laplace’s law (1), for two-layer bandaging at radius 50 mm and bandage width of 100 mm, the optimal tension of the material is 10 N for woven compression bandages (30 mmHg) [41]:

(1) $$P\;(mmHg)=\frac{T\;(N)\cdot n}{r\;(m)\cdot w(m)}\cdot 0.0075$$

where

*P*—pressure;
*T*—tension (applied force);
*n*—number of bandage layers;
*r*—radius of curvature;
*w*—bandage width.

Compression dressings are used in certain cases and require more pressure than standard dressings. Given that FOS is to perform only support functions, it can be assumed that the obtained stress results ensure the mechanical stability of the solution.

### 2.3. Smart Bandage Mobile Application

The presented system is part of the concept of the Internet of Things (IoT). By using an energy-saving radio communication interface (BLE), it was possible to implement two-way communication between the control electronics and mobile devices. In home applications, this type of functionality has become standard; however, in clinical applications, it creates new therapeutic possibilities. The proposed system architecture allows for wide modeling of the functionality of the solution. In practice, it means the possibility of individually tailoring a smart bandage controller, monitoring therapeutic cycles from the mobile device, or modifying the software controlling the smart bandage from a smartphone.

**Figure 9:**
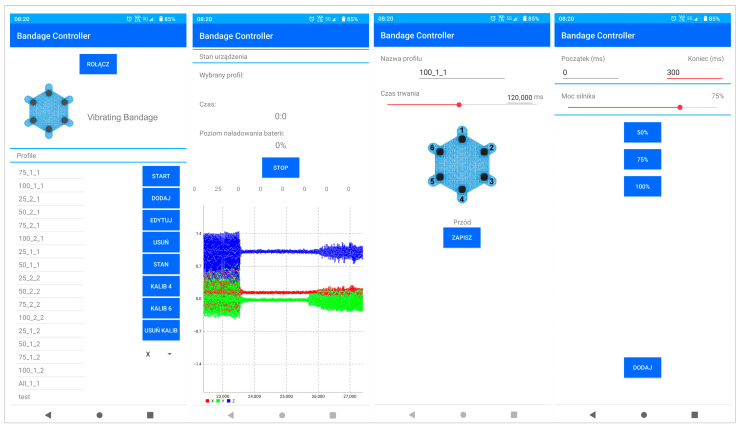
Screenshots from the smart bandage mobile application.

Already at the stage of performed laboratory tests, simple profiles controlling the actuators and auto-calibration process were implemented on the smart bandage electronics side. In practice, it is possible to implement various control profiles in the memory of controllers that can be selected for healing specific types of wounds. As mentioned before, the choice of control profiles can be implemented from the smartphone level or using a switch built into the bandage controller. An important functionality implemented in the mobile software is the ability to develop your own power control schemes for each actuator and the time of their operation. This makes it possible to upload personalized therapeutic profiles, although the practical applications of this functionality require further research. Screenshots from the smart bandage mobile system application are presented in Figure 9.

### 2.4. Calibration Procedure of Actuators

In order to equalize the amplitude of vibrations generated by individual smart bandage vibration actuators, calibration is carried out based on accelerometric measurements. Individual actuators are controlled using a PWM signal. In the calibration procedure, it was assumed that the change in PWM affects a rectilinear increase in generated vibrations. During calibration, each vibration actuator is activated individually, the others are disabled. When the actuator is working, the accelerometer placed in a central bandage pocket carries out measurements with sampling at an ODR (Output Data Rate) of 400 Hz. The calibration procedure begins with a 10% PWM filling, and then the filling increases by 10% until 100% is reached. The first 100 measuring points from the accelerometer are rejected, which corresponds to 250 ms. This time allows the engine to start and stabilize its speed. The accelerometer in the FIFO queue aggregates data into packages, where each data package contains 25 measuring points. A series of 50 packages of measurement data is sent to the microcontroller, where a signal constant component is removed. Then, the absolute value is calculated from the variable component of each measuring point.

In the next stage, the maximum value is selected from the processed data from each FIFO package. Therefore, 50 points are obtained, from which the mean value is calculated. This procedure is used for the measurement data from each actuator with all PWM values. It makes it possible to determine the real characteristics of the averaged oscillation amplitudes (AOA) individually for each actuator. After the measurement cycle and data processing, from the characteristics received for all actuators, the minimum value of AOA is determined with 100% filling and the maximum value of AOA when PWM filling is 10%. These two values allow for determining straight calibration lines for actuators.

The designated straight calibration line contains the expected values of the averaged oscillation amplitudes (AOAs) for the assumed values of the PWM signal filling, which makes it possible to equalize the operation of individual actuators. Then, in the iterative process, the rectangular wave filling is changed until the value of the expected oscillation amplitude is achieved for a specific, assumed filling (from 10% to 100%), and this value for each actuator is saved in the microcontroller memory. The described calibration procedure can be used for various types of actuators and with different power voltage values. Calibration can be performed separately for each of the accelerator axes, and then each component can be analyzed independently. It is also possible to calibrate all the axes at the same time, although the resultant vector of acceleration *a_r_* should be previously determined according to Formula (2):

(2) $$a_{r}=\sqrt{a_{x}^{2}+a_{y}^{2}+a_{z}^{2}}$$

where *a_x_*, *a_y_*, and *a_z_* are the components of acceleration along the *x*, *y*, and *z* axes from the accelerometer.

**Figure 10:**
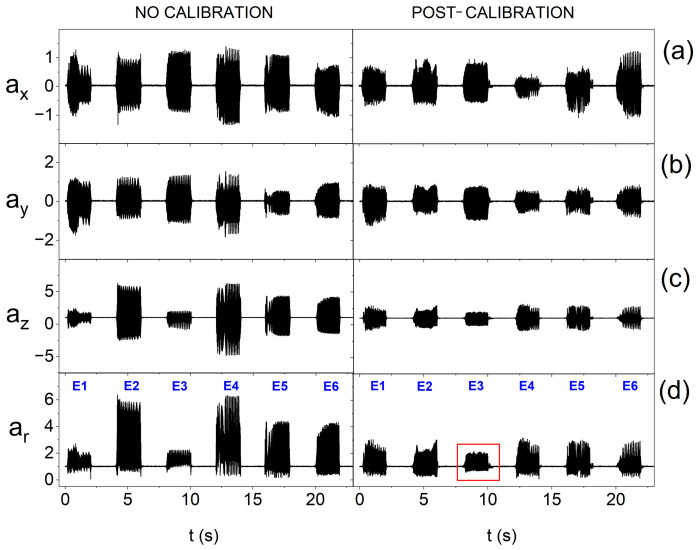
Amplitude of oscillation when placing the smart bandage on the flat surface and supplying actuators with reduced voltage of 1.65 V, before and after calibration, respectively, for the following: (**a**) resultant acceleration vector and its individual components; (**b**) along the x-axis; (**c**) along the y-axis; and (**d**) along the z-axis.

Miniature vibration actuators can differ significantly in terms of parameters, e.g., vibration amplitude in individual axes, which is presented in Figure 10a–c. For this reason, it is more beneficial to calculate the resultant vector, which was used in the measuring procedure. An example of the impact of the calibration procedure on the equalization of oscillations is presented in Figure 10d.

Figure 10d also shows the impact of calibration performed relative to the resultant vector on individual axes. The characteristics of oscillations of individual vibration actuators before calibration (Figure 10d) show a relatively large variety of amplitudes—the weakest actuator generated oscillations that were about 65% weaker than those generated by the strongest one. The use of the calibration procedure makes it possible to reduce the diversity of oscillations to approx. 30%. The calibration procedure also allows for testing the possibility of controlling the vibration amplitudes of individual actuators and selecting subpar systems, e.g., of lower quality or with a greater extent of wear. As mentioned earlier, the smart bandage has a modular structure that allows for quick replacement of the main components, including actuators. It can be observed in Figure 10d that after the calibration process, the amplitude of the resultant acceleration vector *a_r_* of one of the actuators has a lower value (marked with a red rectangle). This may indicate, for example, its lower production quality. In the case of other actuators, the equalization of oscillation amplitudes is visible.

## 3. Results and Discussion

**Figure 11:**
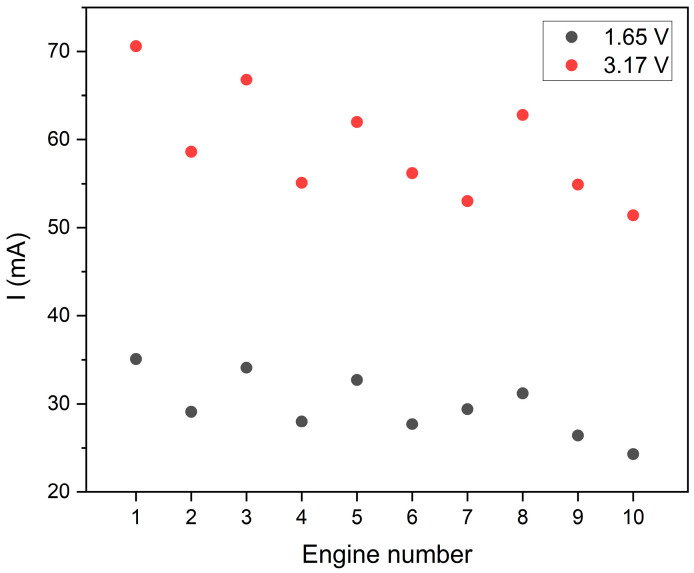
Current consumption by individual oscillators at nominal supply voltage (3.17 V) and at voltage reduced to 1.65 V.

The developed smart bandage system was tested in several configurations. First, the smart bandage operation was verified using various voltages of actuators. To this end, 10 actuators were selected, whose operation was stabilized for 10 s, and then the oscillations they generated were measured. Figure 11 shows the results of current measurements of individual oscillators at a reduced 1.65 V power supply and a nominal supply of 3.17 V. In both cases, the diversity of results was checked, i.e., the ratio of standard deviation to the mean value was calculated. In the case of nominal power (3.17 V), the diversity coefficient was 10.6%, whereas with the lower power supply of 1.65 V, the diversity slightly increased and amounted to 11.6%. In both cases, the obtained values of the diversity coefficient indicate a small scatter of results among individual actuators.

One of the research goals was to verify the possibility of saving energy. Taking into consideration the fact that the energy demand of the system is determined mainly by the actuators, it was verified how effective their operation would be after a relatively high reduction in the supply voltage in relation to the nominal value. The implementation of this type of testing is also interesting in the aspect of controlling the actuators using a voltage change.

By default, the MT24 actuators are powered by 3 V; however, their operation was verified with a power supply reduced to 1.65 V. Tests of this type allow for determining both the current consumption and the range of working voltages of the actuators. Limiting the nominal supply voltage (by almost a half) is beneficial due to the possibility of extending their working time. However, the system stability under such conditions requires verification. Research performed at the supply value of 1.65 V has indicated the lower limit of their use. It should be emphasized that the presented solution is at the conceptual stage. Therefore, this study verified the possibilities of the FOS (made of materials with different stiffness) to distribute vibrations within it.

Verification of the supply voltage range of actuators is also important in the aspect of their efficiency and energy demand. Wearable solutions are mostly battery-powered, and so their working time is associated with the current consumption and capacity of the battery used. The presented solution uses a battery with a nominal capacity of 110 mAh. With the cycle assumed (2 s work and 2 s breaks), within an hour, the engines work for a total of 30 min (the actuators do not work simultaneously but are activated sequentially). With 3 V nominal power supply, the actuator consumes a current of approx. 60 mA (Figure 11). Therefore, the SSB can work for about 3 h. In the estimation, we took into account 80% of the battery capacity provided by the manufacturer (approx. 88 mAh) so as not to overestimate the calculations.

On the other hand, when the actuators are powered by 1.65 V, the mean power consumption is about 30 mA (Figure 11). When using the same battery with a nominal capacity of 110 mAh, the system can cyclically generate vibrations for about 6 h. Studies performed indicate that the MT24 actuators can be controlled at reduced voltage, although this is preferably in combination with an FOS with greater stiffness, e.g., made of nylon. Lowering the supply voltage affects the distribution of vibrations generated within the FOS. Limiting the vibration amplitude, however, does not exclude the energy-saving system operating mode. It should also be emphasized that the value of 1.65 V is not recommended by the manufacturer for the MT24 actuators.

Therefore, assuming that a single vibration therapy session lasts 30 min [33], 10 therapeutic sessions can be performed at 3 V nominal power supply. With a voltage reduced to 1.65 V, 20 sessions can be conducted after charging the battery once.

**Figure 12:**
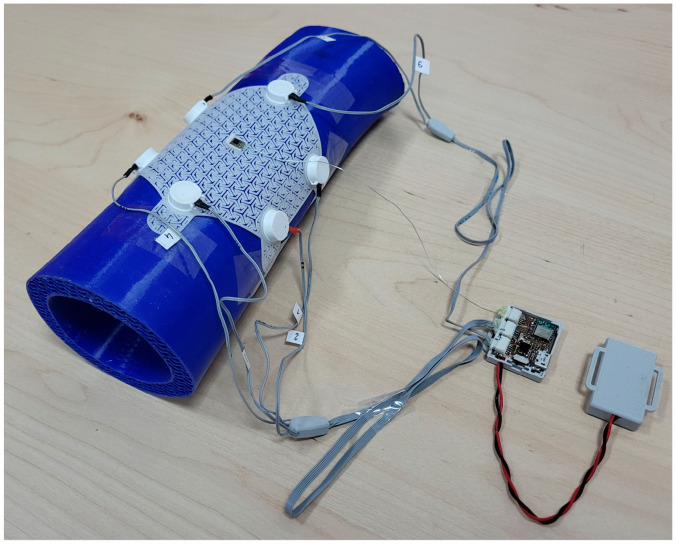
Forearm phantom for testing the smart bandage on the convex surface (on the printed model of the upper limb fragment).

Another analyzed aspect was the fact that wounds may appear in different places on the body and can be of different sizes; therefore, the dressing should be adapted to the geometry of the wound surface. Flat wounds can occur, e.g., on the back or stomach. We deal with convex surfaces, e.g., in the case of limb or head wounds. Therefore, smart bandage tests were planned on two types of surfaces: flat and convex. The PE technical foam was used to model the flat surface. In turn, the convex surface was created using 3D printing. The forearm model was generated and then printed using TPU (thermoplastic polyurethane) material. A view of the phantom forearm is presented in Figure 12. The selection of materials was aimed at simulating the working conditions of a smart bandage for its operation on the human body.

As part of the conducted research, an openwork structure printed from three different materials, i.e., nylon (polyamide), PP (polypropylene), and TPU, was also verified. In this way, structures of various stiffness were obtained. Then, the research procedure took into account the simultaneous impact of the change in the supply voltage of the actuators, the material used for the openwork structure, oscillator calibration, and surface geometry on the smart bandage operation. In all measurements, the actuators were activated sequentially for 2 s with a 2 s interval.

**Figure 13:**
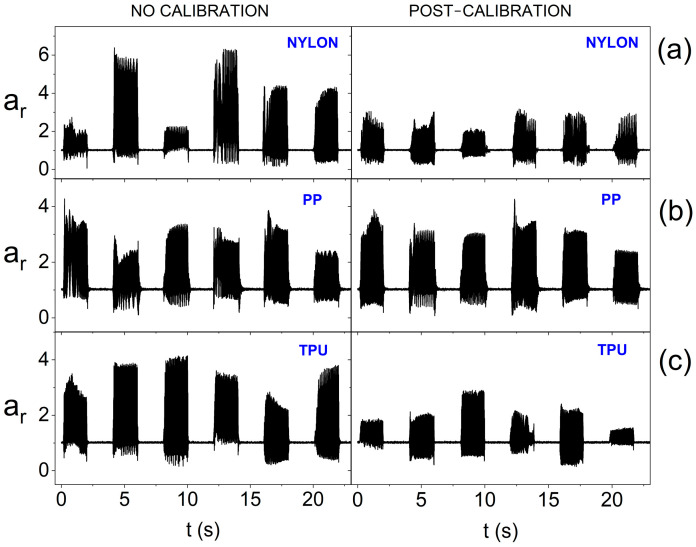
Amplitude of oscillations for the resultant acceleration vector without calibration and after calibration for individual actuators (on the flat surface and at 1.65 V supply), depending on the material from which the openwork structure was made: (**a**) nylon; (**b**) PP; and (**c**) TPU.

Figure 13 shows the impact of the calibration procedure and the material from which the smart bandage openwork structure (FOS) was made when performing tests on the flat surface (PE technical foam) at the reduced power supply of actuators (1.65 V). It can be observed in the chart that the calibration procedure effectively equalizes the vibrations of actuators in the case of the structure made of nylon and PP. However, for the structure made of TPU (the most flexible of the tested ones), a significant diversity of amplitudes for individual actuators is visible, despite the calibration used. Thus, in the case of a flexible openwork structure, a significant reduction in the supply voltage of actuators to reduce energy consumption makes the calibration rather ineffective.

**Figure 14:**
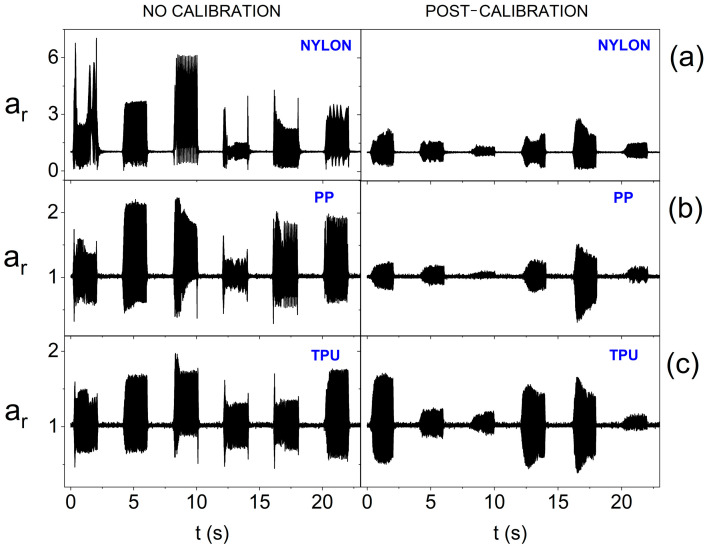
Amplitude of oscillation for the resultant acceleration vector without calibration and after calibration for individual actuators (on the forearm phantom and at 1.65 V supply), depending on the material from which the openwork structure was made: (**a**) nylon; (**b**) PP; and (**c**) TPU.

**Figure 15:**
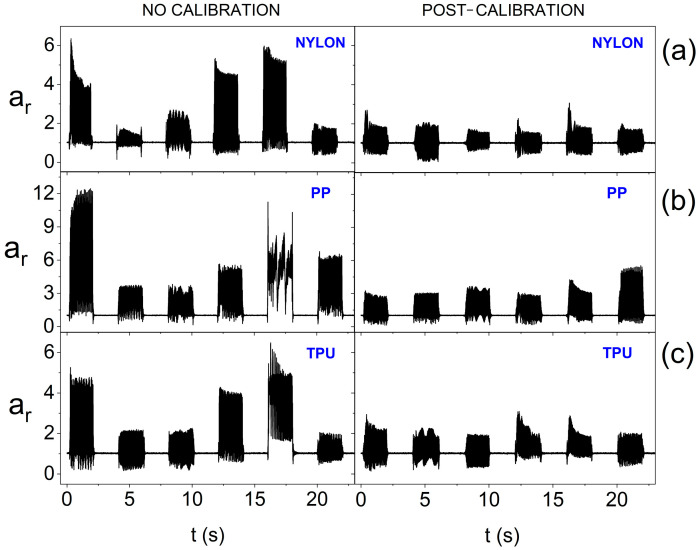
Amplitude of oscillation for the resultant acceleration vector without calibration and after calibration for individual actuators (on the forearm phantom and at 3.17 V supply), depending on the material from which the openwork structure was made: (**a**) nylon; (**b**) PP; and (**c**) TPU.

In turn, Figure 14 also takes into account the surface curvature. Placing actuators, with reduced supply voltage, on a convex surface means that calibration is inefficient for each of the materials used. The smallest diversity of oscillation amplitudes after calibration is noticeable for the stiffest structure, i.e., made of nylon. Therefore, a significant reduction in the power supply of actuators when using the smart bandage on convex surfaces can lead to heterogeneous vibration distribution within its area. Therefore, further tests were performed, in which nominal supply voltage was used, i.e., 3.17 V. The results presented in Figure 15 indicate that increasing the supply voltage value positively affects the homogeneity of the generation of oscillations within FOS. In the case of each of the materials used, equalization of the oscillation amplitudes of individual actuators after calibration is visible.

**Figure 16:**
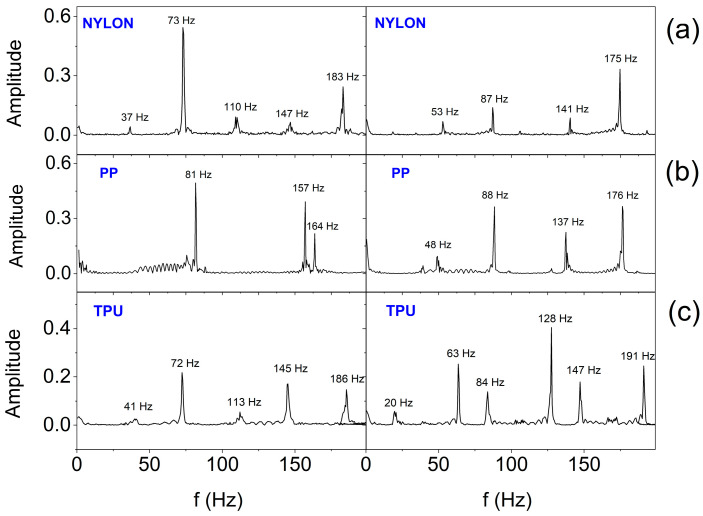
FFT analysis results for two actuators (placed on the flat surface and at 1.65 V supply) depending on the material from which the smart bandage openwork structure was made: (**a**) nylon; (**b**) PP; and (**c**) TPU.

**Figure 17:**
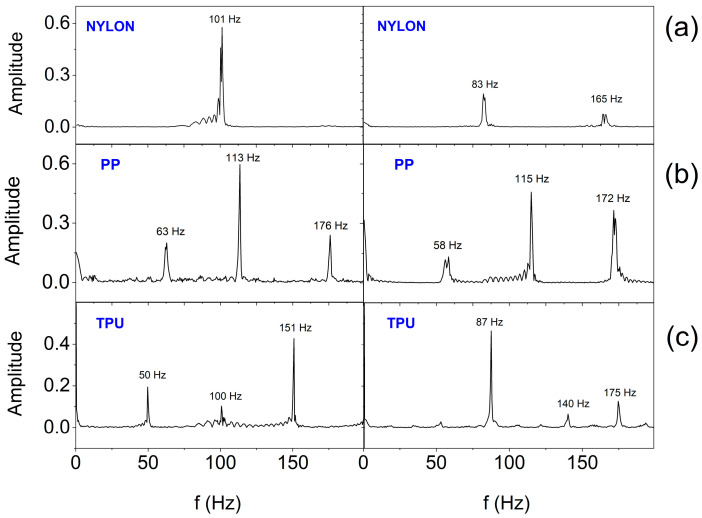
FFT analysis results for two actuators (placed on the forearm phantom and at 3.17 V nominal supply) depending on the material from which the smart bandage openwork structure was made: (**a**) nylon; (**b**) PP; and (**c**) TPU.

In order to estimate the frequency range in which the SSB generates vibrations, Fast Fourier Transform (FFT) analysis was performed. Figure 16 and Figure 17 show the FFT results for the selected two (E2 and E3) out of six actuators working in the smart bandage, appropriately placed on the flat surface and powered with 1.65 V (Figure 16) and placed on the forearm phantom and powered with a nominal voltage of 3.17 V (Figure 17), respectively. In all cases, the operation of actuators was recorded after prior calibration. The analyses performed indicate that the frequency of vibrations ranges from around 40–50 Hz to 180–190 Hz. The vibration amplitude ranges from 0.03 g to 0.6 g. When the smart bandage is placed on the flat surface, more characteristic oscillation frequencies (Figure 17) are generated than when placed on the convex surface (Figure 16). Most likely, when the smart bandage structure bends, mechanical vibrations are filtered within the FOS. Differentiation of the quantity and frequency values of individual peaks, observed for the same oscillators placed in different FOSs, is unlikely to result from the properties of the material from which the structure was printed, although it may be the result of transferring the actuators into the next structure during this research. This situation indicates that the mechanical system (openwork structure–actuator) is relatively complex, if only because of the possibility of clearance fit on matching connections (press fits). The research suggests that satisfactory results can be obtained despite the use of simple mechanical solutions. The results indicate that regardless of the shape of the surface on which the smart bandage is placed, it generates vibrations in the frequency range that is used in vibration therapies. Studies carried out on rats have shown that vibrations with a frequency of 45 Hz can promote the healing process of stage II pressure ulcers [42]. It should be emphasized that current studies on the treatment of chronic wounds in humans (e.g., diabetic neuropathic foot ulcer) indicate the effectiveness of vibrotherapy in the frequency range of about 50 Hz [28].

## 4. Conclusions

This article presents the concept of a smart bandage system made in the widely available 3D printing technology and integrated with high technologies. Despite the simple mechanical structure—which, among other benefits, translates into low costs of producing the hardware base—high functionality has been achieved. This was made possible thanks to combining the system with the concept of the Internet of Things (IoT). As a result, the presented system is characterized by the possibility of personalizing therapy as well as controlling, monitoring, and programming the smart bandage from mobile devices. The modular design of the smart bandage increases its functionality and versatility. The frequency range it generates coincides with the frequencies used in vibrotherapy. In addition, the universality of the solution is supported by the possibility of using various materials for printing the smart bandage openwork structure. Although there were differences in the individual smart bandage configurations, including at different power supply levels of actuators (1.65 V and 3.17 V), it has been shown that the solution can be potentially used for the treatment of wounds on both flat and convex surfaces.

## Data Availability

The original contributions presented in this study are included in the article. Further inquiries can be directed to the corresponding author(s).
